# Motor ability in children treated for idiopathic clubfoot. A controlled pilot study

**DOI:** 10.1186/1471-2431-9-78

**Published:** 2009-12-15

**Authors:** Hanneke Andriesse, Lena Westbom, Gunnar Hägglund

**Affiliations:** 1 Department of Orthopaedics, Lund University Hospital, Sweden, SE-221 85 Lund, Sweden; 2 Division of Paediatrics, Department of Clinical Sciences (Lund), Lund University, Lund, Sweden; 3 Children's Hospital, University Hospital, SE-221 85 Lund, Sweden

## Abstract

**Background:**

To study motor ability at seven years of age in children treated for idiopathic clubfoot and its relation to clubfoot laterality, foot status and the amount of surgery performed.

**Methods:**

Twenty children (mean age 7.5 years, SD 3.2 months) from a consecutive birth cohort from our hospital catchments area (300.000 inhabitants from southern Sweden) were assessed with the Movement Assessment Battery for Children (MABC) and the Clubfoot Assessment Protocol (CAP).

**Results:**

Compared to typically developing children an increased prevalence of motor impairment was found regarding both the total score for MABC (p < 0.05) and the subtest ABC-Ball skills (p < 0.05). No relationship was found between the child's actual foot status, laterality or the extent of foot surgery with the motor ability as measured with MABC. Only the CAP item "one-leg stand" correlated significantly with the MABC (rs = -0.53, p = 0.02).

**Conclusions:**

Children with idiopathic clubfoot appear to have an increased risk of motor activity limitations and it is possible that other factors, independent of the clinical status, might be involved. The ability to keep balance on one leg may be a sufficient tool for determining which children in the orthopedic setting should be more thoroughly evaluated regarding their neuromotor functioning.

## Background

Idiopathic clubfoot is an isolated birth deformity affecting 1:1000 children, with a male-to-female ratio of 2.5:1 [1]. Typically, the forefoot is in inversion including a cavus, the hindfoot is in varus and equinus. Muscle activity is dominated by inversion/plantar flexion. Severity can range from very mild to very severe [2].

Treatment should start as soon as possible after birth. Primarily non-operative management is advocated [2-4] as extensive surgery is often related to inferior outcomes [5]. To maintain the initial correction, treatment continues with the use of an orthosis.

The treatment goal is a foot with sufficient mobility and muscle function enabling daily activity and sport leisure without pain, stiffness or shoe problems. It should be emphasized that a clubfoot can never become "normal". Common findings are a shorter and wider foot, a thinner calf, reduced muscle strength and endurance, and restricted foot and ankle mobility [6,7]. No studies have been made on how these factors influence the child's motor performance. Generally, outcome assessments after clubfoot treatment are focused on joint mobility, radiographs and registration of pain, i.e. items on body structure and body function levels according to the International Classification of Function, Disability and Health (ICF) [8]. Problems in activity and participation domains are sparsely touched. Studies on how children treated for idiopathic clubfoot cope in motor activities are rare [9].

To monitor child movement capacity at our clubfoot clinic, we included the Movement Assessment Battery for Children (MABC) in the routine clinical follow-up at seven years of age. MABC is a standardized screening instrument specifically developed for the evaluation of motor ability in children [10]. We also use a multi-level standardized follow-up instrument specially developed for children treated for clubfoot, the Clubfoot Assessment Protocol (CAP) [11,12]. The CAP includes variables on joint mobility, muscle function, morphology and functional activity (Table 1).

**Table 1:** A summary of the Clubfoot Assessment Protocol (CAP) and its cut-off points.

Items	Scores	Cut-off
**Mobility I**	Total: 0-20	≤ 15
Ankle dorsal extension, plantar flexion, heel varus/valgus, eversion/inversion and forefoot adduction/abduction (5 items)	Item level: 0, 1, 2, 3 and 4	≤ 2
		
**Mobility II**	Total: 0-8	≤ 6
Length of toe flexors (2 items)	Item level: 0, 2 and 4.	≤ 2
		
**Muscle function**	Total: 0-8	≤ 6
Strength of foot extension and eversion (2 items)	Item level: 0, 2 and 4.	≤ 2
		
**Morphology**	Total: 0-16	≤ 12
Tibial torsion, heel and forefoot position, cavus or planus. (4 items)	Item level: 0, 2 and 4.	≤ 2
		
**Motion quality I**	Total: 0-16	<12
Walking, running, toe walking, heel walking (4 items)	Item level: 0, 1, 2, 3 and 4.	≤ 2
		
**Motion quality II**	Total: 0-8	≤ 6
one-leg balance, one-leg hop (2 items)	Item level: 0, 1, 2, 3 and 4.	≤ 2
**CAPtotal (feet level)**	0-76	≤ 57
**CAPtotal (child level)**		≤ 57 (bilateral) ≤ 66.5 (unilateral)

Item level scores: 0 = cannot/++ poor, 1 = very deviant/+ poor, 2 = deviant/poor, 3 = slightly deviant/slightly poor and 4 = within normal.

The aims of this study were to explore motor ability among children treated for idiopathic clubfoot and its relation to clubfoot laterality, foot status and the amount of surgery performed.

### Subjects

Twenty-nine children from our hospital catchments area (300.000 inhabitants from southern Sweden) were consecutively referred for clubfoot treatment between 1995 and 1998. Twenty-two of these new-born children were diagnosed with idiopathic clubfoot and eligible for this study. Written informed consent was obtained from parents of 21 of the 22 children. One child could not be tested at this age because of ongoing postoperative casting and rehabilitation after a tibialis anterior transposition. A total of 20 children (6 girls, 14 boys) with a mean age of 7.5 years (SD 3.2 months) were finally included. Ten had unilateral and 10 bilateral clubfoot. At birth the children showed no statistically significant differences regarding severity between unilateral and bilateral clubfoot according to the Dimeglio classification system [13]. Eighteen feet were classified as moderate and 12 as severe with a median severity score of 10.9 (range 6-15).

All children had been treated according to a modified Copenhagen method [3,9,14]. During weekdays, daily mobilization (stretching of all shortened soft tissue and manipulation of contract joints) was done and weakened muscles were stimulated manually by triggering movement by the physical therapist. Obtained correction was maintained with the use of an adjustable splint. At the age of two months, surgical intervention was performed in 26/30 feet: posteromedial releases including achilles lengthening in 24 feet, and percutaneous achilles tenotomy in 2 feet. Four feet were fully corrected in its varus- adduction component and had a dorsalflexion >5° and continued directly with the orthose treatment. Knee Ankle Foot Orthosis were used during the first two months 18 hours a day, decreasing successively during daytime until the age of 8 months and continued only nighttime until the child started to walk. Thereafter a dynamic Ankle Foot Orthosis was used during nighttime until the age of four years. These orthosis have the ability to give a prolonged stretch to the Achilles tendon. In eight feet a tibialis anterior transfer was performed after the age of three years.

The study was approved by the Ethics Committee at Lund University Hospital (LU 666-03).

## Methods

### Assessment of motor ability

The MABC test contains eight items that represent the main motor skills of children between 4 and 12 years of age. These items are divided into three subgroups: manual dexterity, ball skills and balance, integrating coordination, concentration, visual-spatial perception and balance in fine and gross motor performances. MABC has been proven to be a valid and reliable instrument [10,15,16].

Four age bands are formed with different items but covering similar skills which are age-adjusted (4-6, 7-8, 9-10 and 11-12 years). The total score can vary between 0 and 40 (best-worst). A large sample of norm-reference was studied and the raw scores were transformed into percentiles provided in the manual [10]. An MABC result below the 5th percentile (MABC ≥ 13.5) indicates definite motor problems. Results between the 5th and the 15th percentile (MABC 13.4-10) indicate borderline problems. The motor ability in typically developing children, above the 15th percentile, corresponds to an MABC score below 10.

The MABC subscores and their cut-off percentiles are mainly used for creating a profile on the child's motor difficulties. Factors such as how the child carries out the task (motor quality), the child's behavior (e.g. concentration, impulsivity) and physical deficiencies (vision, neurological and orthopedic problems) have to be incorporated in the evaluation to enable intervention and treatment planning.

In this study only the quantitative data were used and compared to the expected distribution according to the MABC standardization. The children were tested with age band 2 (7-8 years) (Table 2) [10].

**Table 2:** A summary of the Movement ABC (MABC) test age band 2 (7-8 years).

Items	Scores	Cut-off 15th percentile
**Manual dexterity**	Total: 0-15	≥ 5
Placing pegs in a peg board	0-5	
Threading a lace	0-5	
Drawing a continuous line into a trail	0-5	
**Ball skills**	Total: 0-10	≥ 2,5
Bouncing and catching ball with one hand	0-5	
Throwing bean bag into a box	0-5	
**Balance**	Total: 0-15	≥ 5
Stork balance	0-5	
Jumping in squares	0-5	
Heel-to-toe walk on a line	0-5	
**MABC**	Total 0-40	≥ 10

The MABC assessments were done by a neurodevelopmental physiotherapist experienced in the MABC test and with no previous knowledge of the study children or their foot status.

### Assessment of foot status

The CAP (Table 1) was used for analyzing the clinical outcome of the clubfoot treatment. Twenty items divided between body structures/body function and activity domains according to the International Classification of Function, Disability and Health (ICF) [8] form the CAP. The focus is on item and subgroup level. The items scoring range from 0-4 (worst to normal). In previous studies the CAP has been shown to have moderate to good reliability [12] and validity [11].

All CAP assessments were performed by an experienced physiotherapist at the orthopaedic department (HA).

### Procedure

The assessments took place in a child-friendly environment during early afternoon. First the clinical examination was performed, followed by a 15-minute break and finally the MABC test. The whole procedure took approximately one hour.

### Surgery

No surgery or only tendo achilles lengthening (TAL) were categorized as no or minor surgery. Postero-medial release (PMR) or tibialis anterior transfer (TAT) were categorized as extensive surgery.

### Data analysis

Fisher's exact t-test using a multinomial distribution was used to check differences in motor performance between the study group and the distribution in the normal population. Standard Fisher's exact t-test was used to analyze the relationships between surgery and motor capacity and between clubfoot status and motor capacity. Mann-Whitney U-test was used for comparing motor ability between the children with uni- and bilateral clubfoot.

The Spearman correlation coefficient (r_s_) was used to evaluate the correlations between MABC test and the CAP scores. Their graph plots were checked for skewness. The mean values from the right and left foot of CAP assessments were used for correlation between the MABC and the CAP.

For the MABC the 15th percentile was used as cut-off point [15]. As no cut-off points have been established for the CAP to classify good and/or poor overall clubfoot outcomes, we arbitrarily constructed cut-off points for the items, domains and total scores of the CAP (Table 1). The highest total CAP feet score is 76. For children with bilateral clubfeet the cut-off point at child level was calculated as 75% of 76 points = 57 points. For children with unilateral clubfoot the cut-off point at child level was calculated as (76+57)/2 = 66.5 points.

The statistically significant level was set at p < 0.05. Data were analyzed using SPSS 12.0 and StatXact 3.0.

## Results

**Figure 1:**
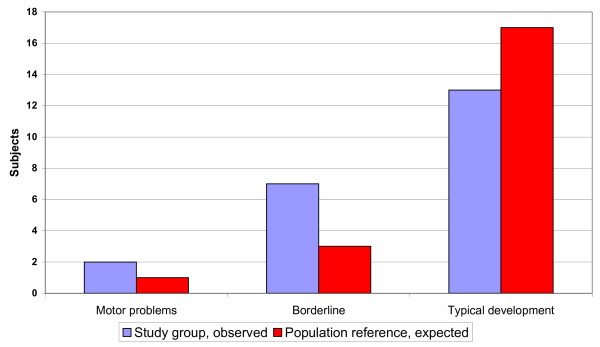


The median MABC score was 6.5, with Inter Quartile Ranges (IQR) 3.6-10.5 (Table 3). Of the 20 children, two had clear motor disability (MABC below the 5th percentile) and five had motor activity limitations (MABC results between the 5th and 15th percentile), compared with the expected one and two respectively (p < 0.05) (Figure 1. Motor ability: Observed versus Expected. Fisher's exact test p < 0.05). No statistically significant relation was seen between the amount of surgery and MABC outcome.

**Table 3:** Median (Inter Quartile Range) for MABC test scores and CAP scores from 20 seven-years old children treated for idiopathic clubfoot.

	Median (IQR)
**Movement ABC**	
Manual dexterity	1.25 (0-4)
Ball skills	0.5 (0.3-4)
Balance	3.0 (2.5-5)
Total score	6.5 (3.6-10.5)
**CAP**	
Mobility I	16 (15-18.8)
Mobility II	8 (6-8)
Muscle function	8 (8-8)
Morphology	14 (12-16)
Motion quality I	13 (12.1-14.6)
Walking	3 (3-4)
Running	3 (3-4)
Toe walking	4 (3-4)
Heel walking	4 (3-4)
Motion quality II	6 (5.5-7.5)
One-leg stand	3 (2.1-4)
One-leg hop	3 (3-4)
Total: feet level	61 (59-68.8)

Children with uni- and bilateral clubfoot showed no statistically significant difference in MABC score (MABC_total _p = 0.49, MABC_Hand function _p = 0.41, MABC_Ball skills _p = 0.87 and MABC_Balance _p = 0.73). In the whole group, significantly increased performance problems at subtest level were found for the MABC-Ball skills (p < 0.05). MABC results below the 15th percentile were present in three of ten children with unilateral and four of ten children with bilateral clubfoot, and in three of six girls and four of 14 boys.

**Figure 2:**
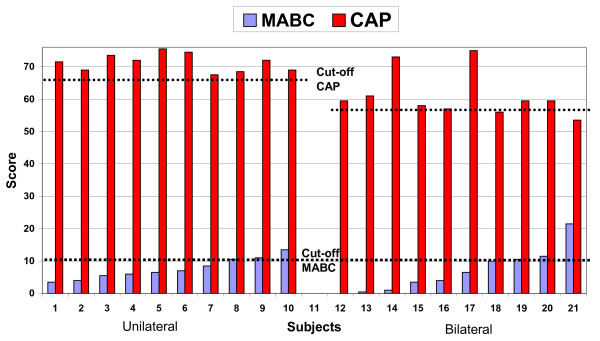


A significant lower (p < 0.05) foot status was found for children with bilateral clubfeet. The median CAP total score at foot level was 67.5 (IQR 62-71) for the children with unilateral clubfoot and 59.5 (55-64) for the children with bilateral clubfoot. At child level the CAP total score showed a median of 71.5 (IQR 69-73.5) for the children with unilateral clubfoot and 59.5 (IQR 57-59.5) for the children with bilateral clubfeet (Figure 2. The relation between motor ability according to the MABC test and the CAP total scores on child level and their cut-off points. The higher the CAP score, the better the foot status; the lower the MABC score, the better the motor activity capacity. Spearman correlation coefficient - 0.24, p > 0.05. Fisher exact test p > 0.05).

Only the item CAP "one-leg stand" showed significant correlations with the MABC-total (- 0.56, p = 0.02).

Three children (all with bilateral clubfoot) had unsatisfactory overall foot status according to the total CAP cut-off points, with a median of 55 (53.7-56.3) (Figure 2). Two of these children had low motor activity capacity in the lowest 15th percentile according to the MABC (21.5 and 10.5 points). None of the ten children with unilateral clubfoot had poor overall status according to CAP, but three of these children showed low motor activity capacity in the 15th percentile on the MABC (13.5, 11 and 10.5 points respectively) (Figure 2).

### Discussion

This is, to our knowledge, the first study performed on motor abilities in an unselected cohort of children of the same age, previously treated for idiopathic clubfoot. In general the clubfoot treatment outcome in this study population was good, with only six (three children with bilateral clubfeet) out of 30 feet showing scores under the cut-off point of the CAP.

A high prevalence of motor activity limitations was found on the MABC testing. Motor ability was not related to clinical foot status, uni- or bilateral clubfoot or amount of surgery performed.

The strengths of this study were that all children were of the same age and assessed by the same assessors (one of whom was blinded), thus increasing reliability. The distribution of uni- and bilateral clubfeet and the gender distribution were as expected [2]. The major limitation of this study is the use of the norm-based MABC percentiles as a reference instead of a matched control group. However, the control groups in the study by Foulder-Hughes LA and Cooke RW [17] and in Davis NM et al [18] showed medians (IQR) of 3.50 (1-6.63) and 1.5 (0.5-3.5) respectively, supporting the norm-based cut-off points of Henderson SE and Sugden A [10]. Others have observed that the MABC norms may be out of date as motor ability might be decreasing in the population [19].

Interestingly, children with unilateral clubfoot had the same degree of motor problems as children with both feet involved, although children with bilateral clubfeet had significantly poorer clubfoot outcome. This indicates that other factors besides foot function play a role in these children's motor abilities. Ball skill problems were significantly more common than expected in this sample, though no high demands are made on the standing ability of the child during these ball tasks.

Balance skill problems according to the MABC showed a tendency to be increased, though not reaching statistical significance in this study of 20 children. Decreased plantar flexion power and mobility are commonly found in treated clubfeet [6,20], and are known factors influencing balance [21]. Primarily, one leg stand is a test specific for postural control incorporating motor control, sensory and cognitive processes. Disturbances in the somatosensory system through surgery may influence perception, a prerequisite for postural control [22]. However, we found no relation between the amount of surgery and MABC outcome.

Expectedly, as static balance is an item in the MABC test, the item CAP **one-leg stand **showed relatively high and significant correlation with the MABC, confirming that they measure similar constructs. No significant correlations were found for the other CAP subgroups, items or total score. We conclude that these two tests mainly assess different functional aspects in children treated for clubfoot. Buus [9] also concluded that the one-leg stand test was the most revealing for the child's neuromotor status.

This study indicates that other dysfunctions may exist in at least a proportion of children with so-called idiopathic clubfoot. Both a high rate of ADHD traits [23] and elevated odd ratios for joint laxity [24] found among children with idiopathic club foot may support this. For several reasons there is an obvious risk that disorders of motor performances in children treated for idiopathic clubfoot might not be recognized or taken seriously. Firstly, outcome instruments used within clubfoot treatment are not focused on performance on activity/participation levels. Secondly, motor disabilities may automatically be related to the child's clubfoot. Particularly in child health surveillance programs, the detection of children with neurodevelopmental disorders may be hampered if deviant motor ability screening results are blamed on the club foot.

Experiencing less success in movement skills, in sports, playground and other daily activities, may cause avoidance of these situations, decreasing the children's opportunities to gain the practice and experience necessary to develop both motor skill competence and social interactions required for well-being [25].

## Conclusions

This study showed that low motor ability as assessed by the MABC was more prevalent in children treated for idiopathic congenital clubfoot but did not automatically relate to the clinical outcome, laterality or the amount of surgery which is commonly assumed. This indicates that other factors beside muscular-skeletal deficiencies might be involved in the child with clubfoot.

The ability to keep balance on one leg may be a sufficient tool for determining which children in the orthopedic setting should be more thoroughly evaluated regarding their neuromotor functioning.

## Competing interests

The authors declare that they have no competing interests.

## Authors' contributions

HA designed the research. HA collected the data and drafted the manuscript. HA, GH and LW analyzed and interpreted the data. GH and LW revised the manuscript. All authors read and approved the final manuscript.

## Pre-publication history

The pre-publication history for this paper can be accessed here:

http://www.biomedcentral.com/1471-2431/9/78/prepub

## References

[B1] Wallander H Hovelius L Michaelsson K Incidence of congenital clubfoot in Sweden Acta Orthop 2006 77 6 847 52 10.1080/17453670610013123 17260191

[B2] Ponseti IV Congenital clubfoot 1996 Oxford: Oxford University Press

[B3] Reimann I Lyquist E Dynamic splint used in the treatment of club foot Acta Orthop Scand 1969 40 817 824 5376792 10.3109/17453676908989546

[B4] Souchet P Bensahel H Theman-Noel C Pennecot G Csukoyi Z Functional treatment of clubfoot: a new series of 350 idiopathic clubfeet with long term follow-up J Pediatr Orthop B 2004 13 189 196 10.1097/00009957-200405000-00009 15083120

[B5] Ippolito E Farsetti P Caterini R Tidisco C Long-term comparative results in patients with congenital clubfoot treated with two different protocols J Bone Joint Surg Am 2002 85-A 7 1286 1294 10.2106/00004623-200307000-00015 12851354

[B6] Cooper DM Dietz FR Treatment of idiopathic clubfoot. A thirty-year follow-up note J Bone Joint Surg Am 1995 77 1477 1489 7593056 10.2106/00004623-199510000-00002

[B7] Dobbs MB Nunley R Schoenecker PL Long-term follow-up of patients with clubfeet treated with extensive soft-tissue release J Bone Joint Surg Am 2006 88 5 986 996 10.2106/JBJS.E.00114 16651573

[B8] International Classification of Function, Disability and Health WHO, Geneva http://www.who.int/classifications/icf/en/

[B9] Buus L Clinical examination of the treatment of children with congenital idiopathic clubfoot Nyt om forskning 2000 9 4 10

[B10] Henderson SE Sugden A Movement Assessment Battery for Children: Manual 1992 The Psychological Corporation, Ltd

[B11] Andriesse H Roos EM Hagglund G Jarnlo GB Validity and responsiveness of the Clubfoot Assessment Protocol (CAP). A methodological study BMC Musculoskelet Disord 2006 15 7 28 10.1186/1471-2474-7-28 PMC1434742 16539716

[B12] Andriesse H Hagglund G Jarnlo GB The clubfoot assessment protocol (CAP): description and reliability of a structured multi-level instrument for follow-up BMC Musculoskelet Disord 2005 18 6 40 10.1186/1471-2474-6-40 PMC1190184 16022741

[B13] Dimeglio A Bensahel H Souchet P Bonnet T Classification of clubfoot J Pediatr Orthop 1995 4 129 316 10.1097/01202412-199504020-00002 7670979

[B14] Stromqvist B Johnsson R Jonsson K Sunden G Early intensive treatment of clubfoot. 75 feet followed for 6-11 years Acta Orthop Scand 1992 63 2 183 8 1590054 10.3109/17453679209154819

[B15] Van Waelvelde H De Weerdt W De Cock P Smits-Engelsman BMC Aspects of the validity of the Movement Assessment Battery for Children Hum Mov Sci 2004 23 49 60 10.1016/j.humov.2004.04.004 15201041

[B16] Smits-Engelsman BCM Fiers MJ Henderson SE Henderson L Interrater reliability of the Movement Assessment Battery for Children Phys Ther 2008 88 286 294 18073266 10.2522/ptj.20070068

[B17] Foulder-Hughes LA Cooke RW Motor, cognitive, and behavioral disorders in children born very preterm Dev Med Child Neurol 2003 45 2 97 103 10.1017/S0012162203000197 12578235

[B18] Davis NM Ford GW Anderson PJ Doyle LW Victorian Infant Collaborative Study Group Developmental coordination disorder at 8 years of age in a regional cohort of extremely low-birth weight or very preterm infants Dev Med Child Neurol 2007 49 5 325 30 10.1111/j.1469-8749.2007.00325.x 17489804

[B19] Hadders-Algra M Atypical performance: how do we deal with that? Dev Med Child Neurol 2007 49 6 403 10.1111/j.1469-8749.2007.00403.x 17518920

[B20] Karol LA Concha MC Johnstone CE Gait analysis and muscle strength in children with surgically treated clubfeet J Pediatr Orthop 1997 17 6 790 795 10.1097/00004694-199711000-00018 9591985

[B21] Nilsson G Ageberg E Ekdahl C Eneroth M Balance in single-limb stance after surgically treated ankle fractures: a 14-month follow-up BMC Musculoskelet Disord 2006 5 7 35 10.1186/1471-2474-7-35 PMC1450283 16597332

[B22] Shumway-Cook A Marjorie H Motor control: theory and practical applications 2001 Baltimore, Maryland: Lippincott Williams & Wilkins11487601

[B23] Max JE Mathews K Manes FF Robertson BAM Fox PT Lancaster JL Schatz A Collings N Attention deficit hyperactivity disorder and neurocognitive correlates after childhood stroke J Int Neuropsychol Soc 2003 9 6 815 829 10.1017/S1355617703960012 14632240

[B24] Olshan AF Schroeder JC Alderman BW Mosca VS Joint laxity and the risk of clubfoot Birth Defects Res A Clin Mol Teratol 2003 67 8 585 90 10.1002/bdra.10085 14632308

[B25] Skinner RA Piek JP Psychosocial implications of poor motor coordination in children and adolescents Hum Mov Sci 2001 20 73 94 10.1016/S0167-9457(01)00029-X 11471399

